# IGF1R signalling in testicular germ cell tumour cells impacts on cell survival and acquired cisplatin resistance

**DOI:** 10.1002/path.5008

**Published:** 2018-01-10

**Authors:** Joanna Selfe, Neil C Goddard, Alan McIntyre, Kathryn R Taylor, Jane Renshaw, Sergey D Popov, Khin Thway, Brenda Summersgill, Robert A Huddart, Duncan C Gilbert, Janet M Shipley

**Affiliations:** ^1^ Sarcoma Molecular Pathology Team, Divisions of Molecular Pathology and Cancer Therapeutics Institute of Cancer Research London UK; ^2^ Glioma Team, Divisions of Molecular Pathology and Cancer Therapeutics Institute of Cancer Research London UK; ^3^ Sarcoma Unit, Department of Histopathology The Royal Marsden NHS Foundation Trust London UK; ^4^ Department of Clinical Oncology The Royal Marsden NHS Foundation Trust Sutton UK; ^5^ Sussex Cancer Centre Royal Sussex County Hospital Brighton UK

**Keywords:** testicular germ cell tumours, nonseminomas, IGF1R, receptor tyrosine kinase

## Abstract

Testicular germ cell tumours (TGCTs) are the most frequent malignancy and cause of death from solid tumours in the 20‐ to 40‐year age group. Although most cases show sensitivity to cis‐platinum‐based chemotherapy, this is associated with long‐term toxicities and chemo‐resistance. Roles for receptor tyrosine kinases other than KIT are largely unknown in TGCT. We therefore conducted a phosphoproteomic screen and identified the insulin growth factor receptor‐1 (IGF1R) as both highly expressed and activated in TGCT cell lines representing the nonseminomatous subtype. IGF1R was also frequently expressed in tumour samples from patients with nonseminomas. Functional analysis of cell line models showed that long‐term shRNA‐mediated IGF1R silencing leads to apoptosis and complete ablation of nonseminoma cells with active IGF1R signalling. Cell lines with high levels of IGF1R activity also showed reduced AKT signalling in response to decreased IGF1R expression as well as sensitivity to the small‐molecule IGF1R inhibitor NVP‐AEW541. These results were in contrast to those in the seminoma cell line TCAM2 that lacked IGF1R signalling via AKT and was one of the two cell lines least sensitive to the IGF1R inhibitor. The dependence on IGF1R activity in the majority of nonseminomas parallels the known role of IGF signalling in the proliferation, migration, and survival of primordial germ cells, the putative cell of origin for TGCT. Upregulation of IGF1R expression and signalling was also found to contribute to acquired cisplatin resistance in an in vitro nonseminoma model, providing a rationale for targeting IGF1R in cisplatin‐resistant disease. © 2017 The Authors. *The Journal of Pathology* published by John Wiley & Sons Ltd on behalf of Pathological Society of Great Britain and Ireland.

## Introduction

Testicular germ cell tumours (TGCTs) are the most common solid malignancy in young adults and adolescents. They comprise two major histological subtypes: seminomas, which resemble primordial germ cells (PGCs), the likely precursors of TGCT; and nonseminomas, which display features of embryonic, extra‐embryonic, and somatic tissue. TGCTs are thought to originate through improper development of PGCs and/or gonocytes (reviewed in ref 1). Signalling through the KIT receptor tyrosine kinases (RTKs) is important in this process, as this, and more recently IGF1R, has been shown to promote the motility and survival of PGCs 2, 3, 4.

Over 95% of patients presenting with TGCT are cured, either through orchidectomy at an early stage or by the effective use of combination cisplatin‐based chemotherapy 5. Patients who present with poor risk features (extremely high tumour markers, non‐pulmonary visceral metastases or mediastinal primary tumour) have survival rates below 70% and despite initial favourable responses, cisplatin resistance can develop 6. The typically young age at diagnosis means that, on average, approximately 36 years of life are lost per man dying of TGCT from an average 81.5 years' life expectancy in the UK 6. Overcoming chemo‐resistance is key to improving survival in poor prognosis patients.

Inhibition of RTKs has realized their therapeutic potential in a range of cancers over the past decade. Humanized monoclonal antibodies such as trastuzumab (ERBB2 inhibitor) and bevacizumab (VEGF inhibitor) successfully target either the RTK or its ligand to block downstream signalling. Small‐molecule inhibitors of intracellular RTK signalling also have proven activity in many tumour types [e.g. imatinib and gastrointestinal stromal tumour (GIST), sunitinib and renal cell carcinoma, and erlotinib in non‐small cell lung cancer 7, 8, 9]. Previous work by us and others has shown the involvement of RTK signalling in seminomas (SEs) by showing that the gene encoding KIT is amplified (21% of SEs) 10, 11, expressed (78–100% of SEs) 12, 13, 14, 15, and mutated (10–25% of SEs), together suggesting a strong selection pressure for KIT signalling in SE 16, 17, 18. A reduction in viability in response to siRNA‐mediated silencing of KIT in SE cells suggests dependency in this cell type 19.

There is growing evidence that indicates a role for activation of the RAS, MAP kinase, and PI3‐kinase pathways in both the seminoma and the nonseminomatous subtypes of TGCT 20, 21, 22. Downstream elements of RTK signalling activated in TGCT include genomic gain of RASGEF1A and loss of inhibitory factors such as PTEN 20 and PIK3IP1 22. Gain of 12p is a hallmark of TGCT and the minimum region of amplification almost invariably includes the *KRAS* gene 10, 23. Upregulation of RAS signalling occurs through activating mutations of *RAS* genes (*KRAS*, *NRAS*, and *HRAS*) with a combined frequency of 8% 24, 25. Activating *BRAF* mutations are reported in 9% of nonseminomas and have been linked with a poorer prognosis 25, 26. However, ERK is constitutively active in TGCT, irrespective of the mutation status of upstream signalling components *KIT*, *RAS* or *BRAF* 16, 25. The PI3‐kinase pathway is activated via loss of heterozygosity (36%), inactivating mutations (9%), and decreased expression of the PI3‐kinase inhibitor PTEN (72%) in TGCT 20. Additionally, genome‐wide association studies have implicated polymorphisms in downstream effectors of RTKs in predisposition to TGCT 1, 27. Together, there is strong evidence for upregulation of pathways known to signal downstream of RTKs in both the seminoma and the nonseminoma subtypes of TGCT. However, evidence for which RTKs contribute to TGCT behaviour, beyond KIT in seminomas, is limited.

The aim of this study was to identify a role for specific RTK activity in TGCT. We first screened TGCT cell lines for RTK phosphorylation and identified IGF1R as phosphorylated in TGCT. High IGF1R activity was confirmed in TGCT samples and the dependence on IGF1R activity was demonstrated in nonseminoma cell line models, including a model of cisplatin resistance.

## Materials and methods

### Cell lines and tissue samples

The human cell lines GCT27, 577MF, TERA1, NTERA2, 2102 (2102EP), and GCT44 all represent nonseminomas (TCAM2 resembles seminomas) and were cultured as described previously 28, 29, 30, 31, 32, 33. SuSa (cisS), a human teratoma‐derived cell line, and its cisplatin‐resistant subline SuSa cisR have been described previously 34, 35. Samples were collected from TGCT patients (summarized in the supplementary material, Table S1) at the Royal Marsden Hospital NHS Foundation Trust. Informed consent was obtained from all patients; the study was approved by the Institute of Cancer Research/Royal Marsden Hospital Committee for Clinical Research (study number CCR2014) and procedures were in accordance with the Helsinki Declaration. Total RNA was extracted from frozen TGCT samples as described previously 24. Normal tissue RNAs (smooth muscle, lung, uterus, skin, ovary, adipose, brain, stomach, bladder, thymus, placenta, thyroid, small intestine, spleen, pancreas, colon, bone marrow) were obtained from BD Biosciences (Palo Alto, CA, USA). TGCT formalin‐fixed, paraffin‐embedded tissues [see below for immunohistochemistry (IHC) and associated tissue microarrays (TMAs)] have been described previously 36.

### Phospho RTK screen

The Human Phospho‐RTK Array Kit (ARY001) (R&D Systems, Abingdon, UK) was used according to the manufacturer's recommendations. This antibody‐based technique simultaneously determines the levels of phosphotyrosine in 42 RTKs per sample. 5 × 10^6^ cells grown in normal serum were harvested and pelleted, and then resuspended in 500 μl of NP‐40 lysis buffer and precleared. Lysates were quantified using the Bio‐Rad DC Protein Assay (Bio‐Rad, Hemel Hempstead, UK).

Five hundred micrograms of cell line protein was added to each array and incubated for 16 h at 2–8 °C before washing according to the manufacturer's instructions. 1.5 ml of freshly diluted (1:2000) Anti‐Phospho‐Tyrosine‐HRP Detection Antibody was added to each well and incubated for 2 h at room temperature. Arrays were developed using ECL Plus (GE Healthcare, Amersham, UK) according to the manufacturer's instructions and activity was detected using the Molecular Imager ChemiDoc XRS System (Bio‐Rad). The signal intensity was independently assessed on a grading system of 0–5 by three individuals and the consensus score was used (as described previously 37). Each blot was also analysed by densitometry and each RTK intensity was expressed as a fraction of the total signal on the membrane. The ranking of RTK signal intensity by each method was highly similar.

### Phospho‐IGF1R ELISA

Cells were either unstimulated or stimulated with IGF‐1 (Peprotech EC Ltd, London, UK) or IGF‐2 (Peprotech EC Ltd) at 50 ng/ml in normal serum for 5 min prior to lysis. Cell lysates were quantified using the BCA Protein assay (Thermo Fisher Scientific, Waltham, MA, USA). The phospho‐IGF1R ELISA assay (R&D Systems) was used according to the manufacturer's instructions using 100 μg of protein per well. Absorbance at 450 nm was measured on an ELx800 Absorbance Microplate Reader (BioTek, Swindon, UK).

### Immunoblotting

Protein was extracted from cell lines using lysis buffer [Cell Signaling Technology (CST), Leiden, The Netherlands]. Lysates were transferred onto polyvinylidene difluoride membrane using the iBlot system (Thermo Fisher Scientific) according to the manufacturer's instructions. Antibodies {IGF1R (3027; CST), phospho‐AKT (9271, Ser473; CST), AKT (9272; CST), phospho‐IGF1R (Tyr1316) (6113; CST), phosphotyrosine (4G10; Merck Millipore, Watford, UK), phospho‐ERK (Thr202/Tyr204) (9107; CST), ERK (9102; CST), phospho‐STAT3 (Tyr705) (9145; CST), STAT3 (9139; CST), TUBA1A (α‐tubulin) [sc‐8035; Santa Cruz Biotechnology (SCBT), Dallas, TX, USA], ACTB (sc‐47778; SCBT) and GAPDH (MAB374; 1:10 000; Merck Millipore)} were diluted 1:1000 unless otherwise stated in 1% milk–TBST and blots were incubated overnight at 4 °C with agitation. Antibody staining was detected with ECL Prime reagent (GE Healthcare), according to the manufacturer's instructions. Band intensities were digitally measured using ImageJ software 38, 39.

### Immunoprecipitation

Five hundred micrograms of whole cell lysate was adjusted to 1 μg/μl in cell lysis buffer and supplemented with activated sodium vanadate (final concentration 40 mm). IGF1R antibody (1:50 dilution; CST) or normal rabbit IgG (1 μg; SCBT) was added to diluted lysates and rotated overnight at 4 °C. Dynabeads Protein A (50 μl; Thermo Fisher Scientific) were added to lysates and rotated at 4 °C for 3 h. The supernatants were then removed and the beads washed according to the manufacturer's instructions. Immunoprecipitated proteins were eluted by placing beads at 70 °C in 20 μl of 1× LDS Sample Buffer, 50 μm dithiothreitol (DTT; Thermo Fisher Scientific). The eluates were then subjected to immunoblotting.

### Immunohistochemistry

Previously described TMAs 36 were deparaffinized and immunostained with a primary antibody for IGF1R (#3027; CST). Staining was performed on the Leica Bond III platform using Bond Polymer Refine DAB detection (Leica Biosystems, Newcastle‐upon‐Tyne, UK). Antibody was applied at a dilution of 1/200 for 15 min at room temperature, following on‐board heat‐mediated epitope retrieval using Leica Epitope Retrieval 2 solution (pH 9) for 30 min at 99 °C (performed by University College London Advanced Diagnostics). Colon tissue sections were used as controls where discrete specific membranous staining of glandular cells was observed as expected. Slides were analysed by pathologists (SP and KT) and samples with at least two scorable cores were scored positive if more than 10% of cells showed membranous staining.

### RT‐qPCR

cDNA was produced from RNA using Superscript II reverse transcriptase (Thermo Fisher Scientific) according to the manufacturer's instructions. Real‐time RT‐PCR was performed using the ABI PRISM 7900 Sequence Detection System according to the manufacturer's instructions (Thermo Fisher Scientific). Pre‐designed primer and probe sets for test (IGF1R Hs99999020_m1) and control (B‐Actin 4310881E‐0803023) genes were used (Thermo Fisher Scientific). Expression was averaged across triplicate repeats, quantified against a standard curve.

### Copy number analysis

Copy number analysis was carried out using normal male genomic DNA as a calibrator (Promega, Southampton, UK) by real‐time PCR on the Viia7 instrument (Thermo Fisher Scientific) according to the manufacturer's instructions, using IGF1R copy number assay (Hs05381099_cn) and internal reference control gene (RPPH1) assay (Thermo Fisher Scientific). All reactions were performed in triplicate.

### siRNA knockdown of target genes

Transfections were carried out according to the manufacturer's instructions, using Lipofectamine 2000 (Invitrogen, Carlsbad, CA, USA), siRNA duplexes that targeted *IGF1R* (Hs_IGF1R_1 HP, Hs_IGF1R_7 HP) (Qiagen, Hilden, Germany), and a non‐targeting control siRNA (Dharmacon, Lafayette, CO, USA) at 33 nm. Each transfection included six replicates.

### Lentiviral transfection

shRNA sequences targeting IGF1R (MISSION^®^ TRC shRNA TRCN0000000424; Sigma‐Aldrich, Poole, UK) and a non‐targeting control (SHC002; Sigma‐Aldrich) were used in lentiviral shRNA knockdown. Lentivirus production and transduction were as previously described 40. Two days post‐transduction, cells were selected in either 2 μg/ml (NTERA2, GCT44, SuSa) or 5 μg/ml (TCAM2) puromycin.

### Proliferation

Cellular proliferation was assessed using a CyQUANT NF Cell Proliferation Assay Kit (Thermo Fisher Scientific) according to the manufacturer's instructions. Fluorescence intensity was measured (excitation at 485 nm, emission at 535 nm) using a VICTOR2 D fluorometer (PerkinElmer, Beaconsfield, UK). Cells were counted directly using a haemocytometer following lentiviral experiments.

### GI_50_ assays

Cells were plated at 4000 cells per well in a 96‐well plate. The following day, media were replaced with media containing NVP‐AEW541 (Selleck Chemicals; Stratech Scientific, Newmarket, UK) using DMSO as a carrier control (0.1%). Cells were incubated for 72 h before being assayed for viability using the CellTiter Aqueous One Solution Cell Proliferation Assay (Promega) following the manufacturer's instructions. Absorbance at 490 nm was measured on an ELx800 Absorbance Microplate Reader (BioTek).

### Caspase‐Glo 3/7 apoptosis assay

Quantitation of caspase 3/7 activity using the Caspase‐Glo 3/7 Assay (Promega) was performed according to the manufacturer's instructions. Parallel cultures were counted using a haemocytometer to account for discrepancies in cell number between samples. The signal was quantified using an MLX luminometer (Dynex Technologies, Worthing, UK).

## Results

### Phosphorylation RTK screen identifies IGF1R as activated in TGCT

Seven TGCT cell lines, including six nonseminomas and one seminoma, were profiled using antibody arrays to determine the major RTKs activated (supplementary material, Figure S1). The average over all cell lines was used to rank the activity of the RTKs (Table 1). IGF1R was the RTK with the most activity, five out of seven lines showing high levels of phosphorylation. The insulin receptor, which can heterodimerize with IGF1R, also demonstrated high levels of phosphorylation in a similar pattern to IGF1R, consistent with the concept that the IGF pathway is active in TGCT.

**Table 1:** RTK phosphorylation in TGCT cell lines using phospho arrays

	**Cell line**						
**Antibody**	**GCT27**	**GCT44**	**2102**	**577MF**	**TERA1**	**TCAM2**	**NTERA2**
IGF1R	2	3	2	5	3	4	4
INSR	2	3	2	4	3	3	3
TYRO3	3	2	3	3	3	3	3
EPHB2	2	2	2	2	2	2	2
EGFR	2	1	1	2	N	2	3
PDGFRB	0	2	2	1	3	1	3
ROR1	2	1	2	1	2	2	2
MSPR	1	1	2	2	2	1	2
ROR2	1	1	1	2	3	1	1
MERTK	1	1	1	2	N	1	2
TEK	1	1	1	2	N	1	2
FGFR2	0	1	1	2	3	0	1
VEGFR2	0	1	1	2	3	0	1
ERBB4	1	1	1	1	2	0	1
FGFR3	0	1	1	1	1	1	2
RET	1	1	1	1	2	0	1
NTRK2	1	1	1	1	2	0	1
EPHA1	1	1	1	1	2	0	1
ERBB3	1	2	0	1	2	0	0
TIE1	1	1	1	1	1	0	1
VEGFR3	0	1	1	1	1	0	2
EPHA2	1	1	0	2	1	0	1
EPHA4	1	1	0	3	1	0	0
EPHA7	0	0	1	1	N	0	3
PDGFRA	0	1	1	1	1	0	1
FLT3	0	1	1	1	1	0	1
CSF1R	0	1	1	1	1	0	1
EPHB6	0	0	1	1	1	1	1
ERBB2	1	1	0	1	N	0	1
NTRK1	0	1	1	1	N	0	1
AXL	0	0	0	1	0	0	3
VEGFR1	0	1	1	1	0	0	1
EPHB1	0	1	0	1	1	0	1
EPHB4	0	0	1	1	1	0	1
FGFR1	0	0	1	1	1	0	0
NTRK3	0	0	0	1	1	0	1
MUSK	0	0	0	1	1	0	1
FGFR4	0	0	0	1	1	0	0
KIT	0	1	0	0	0	0	1
EPHA3	1	0	0	1	0	0	0
MET	0	0	0	0	N	0	0
EPHA6	0	0	0	0	N	0	0

The phosphorylation of each RTK spotted onto the phospho array represented by signal density was assessed on a scale of 0–5, 0 being no detectable signal and 5 being a very strong signal. The average score across all cell lines was used to determine the overall ranking of RTK phosphorylation. N indicates that the signal was unable to be assessed for this cell line, due to high local background.

### Validation of IGF1R phosphorylation in TGCT cell lines

**Figure 1:**
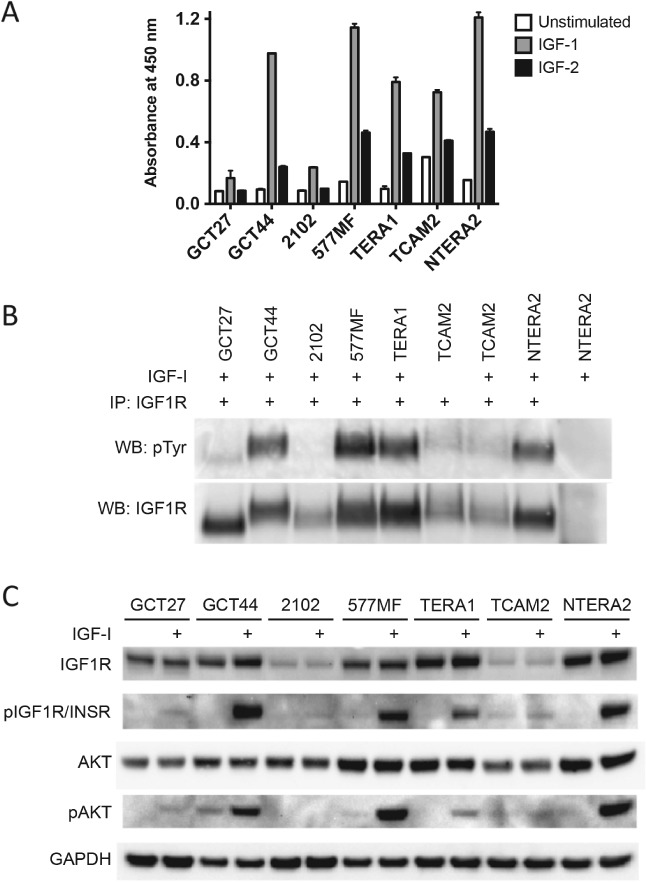
Phospho‐IGF1R levels in TGCT cell lines in response to IGF‐1 and IGF‐2 ligand. (A) Phospho‐IGF1R levels were independently assessed by ELISA in cells (in normal serum) unstimulated or stimulated with 50 ng/ml IGF‐1 or IGF‐2 for 5 min. (B) Immunoprecipitation and consequent immunoblotting with anti‐phosphotyrosine were also carried out with unstimulated or IGF‐1‐stimulated cells. (C) Activated levels of IGF1R/INSR and AKT were also assessed in all cell lines by immunoblotting. Densitometry data corresponding to these blots are shown in the supplementary material, Figure S3.

Two approaches were used to substantiate high IGF1R activity. Phosphorylation of IGF1R in TGCT cell lines was initially confirmed using a phospho‐IGF1R ELISA assay including response to IGF1R ligands IGF‐1 and IGF‐2 (Figure 1A), followed by immunoprecipitation (IP) of IGF1R and immunoblotting with anti‐phosphotyrosine antibodies (Figure 1B). The ELISA and immunoprecipitation assays indicated that unstimulated phospho‐IGF1R levels were generally low, with the only seminoma cell line, TCAM2, having detectable levels by IP. However, four of the six nonseminoma cell lines (GCT44, NTERA2, TERA1, and 577MF) showed a robust response to IGF‐1 and IGF‐2 stimulation by ELISA and IP. Upon IGF‐1 stimulation, TCAM2 showed moderate pIGF1R levels by ELISA but no increase by IP. GCT27 and 2102, which had the lowest pIGF1R levels on the RTK arrays, showed little or no increase in activity when stimulated by IGFs using either method.

### IGF1R activity induces AKT signalling

The levels of pAKT induction when the IGF1R pathway is activated were also investigated (Figure 1C). GCT44, NTERA2, and 577MF showed high levels of pAKT in response to IGF‐1 stimulation, whereas TERA1 showed a more modest induction. pAKT was undetectable in 2102 cells and did not increase with IGF‐1 stimulation in TCAM2 cells. Although GCT27 cells had low amounts of pIGF1R, an increase in pAKT levels was observed with IGF‐1 stimulation. Together, these results support IGF pathway activity and response to ligand in four out of six nonseminoma cell lines, which was not seen in the seminoma cell line.

### IGF1R is highly expressed in normal testes and clinical TGCT samples

**Figure 2:**
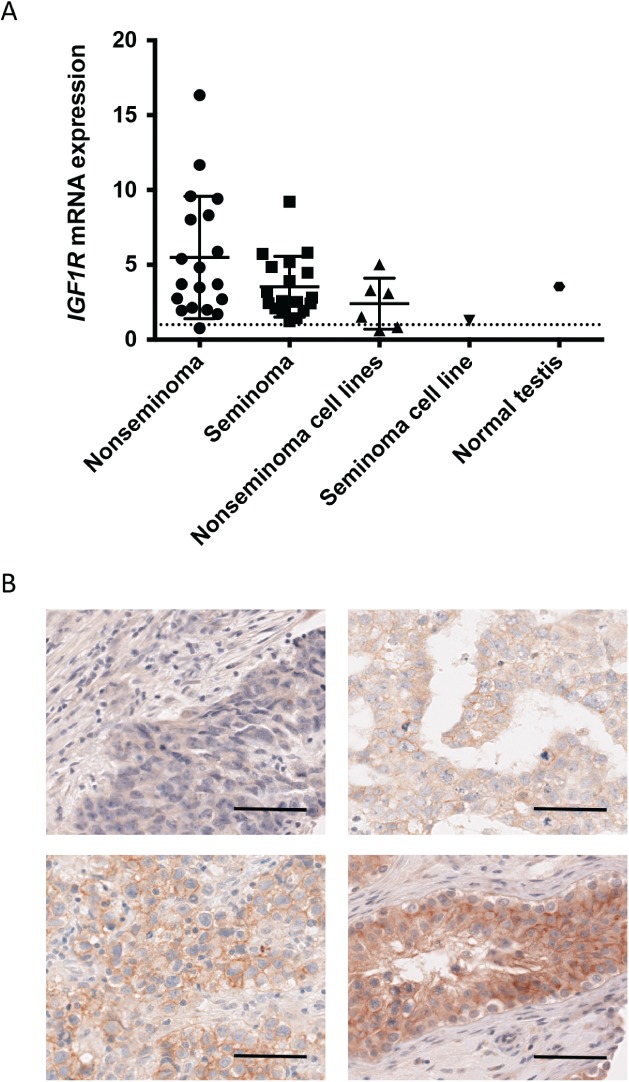
IGF1R expression at protein and mRNA level in primary TGCT samples. (A) IGF1R mRNA levels in the two histological subtypes of TGCT, cell lines, and normal testis as compared to the median level in a panel of normal tissues (set to 1 and indicated by a dashed line) as assessed by RT‐qPCR. (B) Representative images of IGF1R expression in nonseminomas as assessed by IHC. Negative expression (top left), weak expression (top right), moderate expression (bottom left), and strong expression (germ cell neoplasia in situ case, bottom right). Scale bar indicates 50 μm.

The expression level of IGF1R was analysed in tumour samples by RT‐qPCR. Expression of IGF1R was significantly higher in both nonseminomas and seminomas than in normal tissues (p = 0.005 and p = 0.013, respectively; two‐tailed t‐test with Welch's correction) (Figure 2A). However, the difference between nonseminomas and seminomas did not reach statistical significance (p = 0.0768). The median expression of IGF1R in normal tissues was considerably lower than in normal testis (0.28‐fold, data not shown), indicating that IGF1R expression is relatively high in normal testis. All but one nonseminoma sample demonstrated higher IGF1R expression than the median of the normal tissue panel.

IGF1R expression in TGCT was also investigated by immunohistochemistry on tissue microarrays representing 178 cases. Overall, 73/148 (49%) nonseminomas expressed IGF1R (Figure 2B) and the majority of seminomas and germ cell neoplasia *in situ* cases were positive (Table 2, Figure 2B, and supplementary material, Table S2). A chi‐squared test for trend analysis to detect a difference between staining in nonseminomas and seminomas approached significance (*p =* 0.0531).

**Table 2:** IGF1R staining of TGCT primary tumour TMAs

TMA	Histological subtype	No of tumours scored	No of tumours with IGF1R expression	Percentage of tumours with IGF1R expression (%)
TMA 1	GCNIS	14	11	79
	Seminoma	16	13	81
	Nonseminoma	82	34	41
TMA 2	Stage 1 nonseminoma	66	39	59
Total	Nonseminoma	148	73	49
Total	All subtypes	178	97	54

GCNIS: germ cell neoplasia *in situ*.

### IGF1R knockdown reduces growth and causes apoptosis in nonseminoma cells

**Figure 3:**
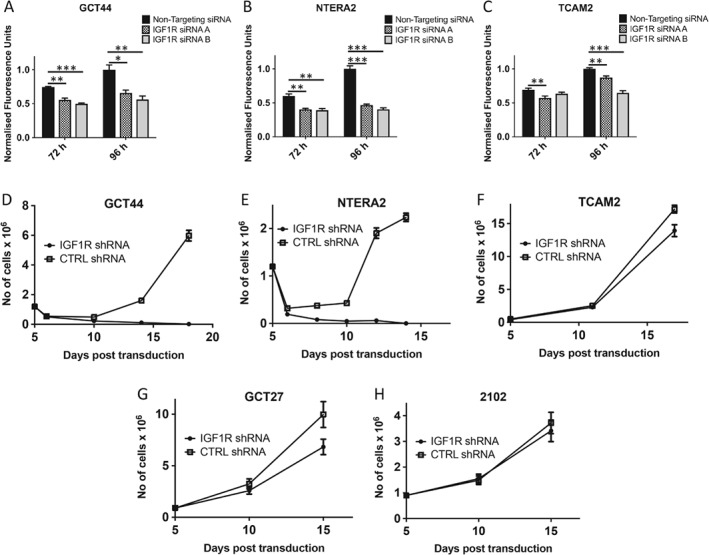
Silencing of IGF1R reduces growth in nonseminoma cell lines. Nonseminoma cell lines GCT44 (A) and NTERA2 (B) exhibited reduced growth upon IGF1R knockdown by two independent siRNAs, whereas the seminoma cell line TCAM2 showed only modest growth retardation (C). Following lentiviral transduction of IGF1R shRNA and selection, the nonseminoma cell lines GCT44 (D) and NTERA2 (E) were eliminated by day 18 of culture in contrast to the seminoma cell line TCAM2 (F) and 2102 (H), which demonstrated a small reduction in cell number after 17 days in culture; GCT27 (G) showed an intermediate response.

The functional consequences of silencing IGF1R expression were assessed in cell lines. NTERA2 and GCT44 were chosen as model nonseminoma cell lines as they both have high activation of IGF1R. siRNA knockdown with two different siRNAs resulted in a strong reduction in viability in both NTERA2 (45% reduction at 96 h post‐transfection) and GCT44 (60% reduction at 96 h post transfection) relative to the non‐targeting siRNA (Figure 3A–C and supplementary material, Figure S2A) in comparison to a milder phenotype observed in TCAM2 (25% reduction at 96 h post‐transfection).

Lentiviral IGF1R shRNAs were used to study the long‐term effects of IGF1R knockdown. The growth of both nonseminoma cell lines was severely affected after shRNA‐mediated reduction of IGF1R. By day 17 post‐transduction, IGF1R‐silenced cells of NTERA2 and GCT44 were dead in comparison to the actively growing cells transduced with a control shRNA (Figure 3D, E and supplementary material, Figure S2B). On day 8 post‐transduction, the cells with IGF1R knockdown were undergoing apoptosis, as shown by a significant increase in the caspase 3/7 activity (p < 0.05) in NTERA2 cells (supplementary material, Figure S2C). In contrast, TCAM2, GCT27, and 2102 cells continued to actively divide with IGF1R silencing, albeit at a retarded rate compared with the control shRNA‐containing cells, with GCT27 cells demonstrating the largest proliferative difference and 2102 cells the smallest (Figure 3F–H). This is consistent with the proliferative phenotype observed after siRNA knockdown of IGF1R, where IGF1R knockdown had a greater impact on the number of viable NTERA2 and GCT44 cells relative to the non‐targeting control and TCAM2 had the mildest phenotype.

### IGF1R silencing leads to attenuated phospho‐AKT signalling in nonseminoma cells and correlates with sensitivity to the IGF1R inhibitor NVP‐AEW541

**Figure 4:**
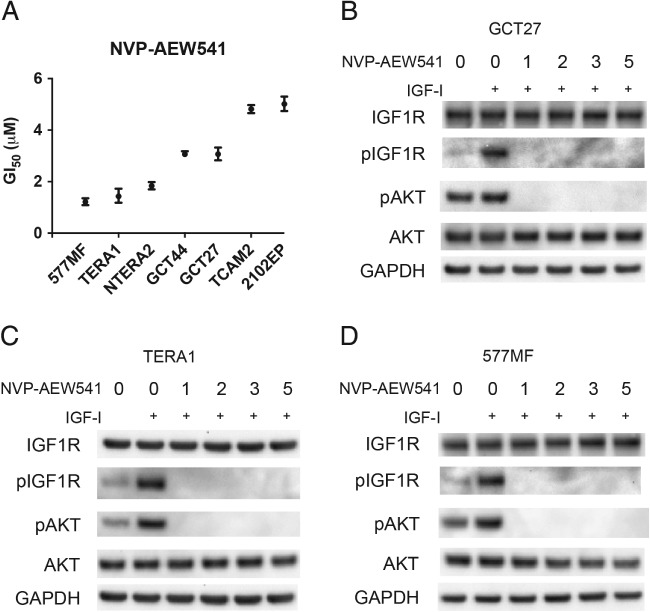
Sensitivity of TGCT cell lines to the IGF1R inhibitor NVP‐AEW541. (A) The GI_50_ of TGCT cell lines to NVP‐AEW541 was measured following 72 h exposure of varying concentrations of inhibitor (in normal serum). (B–D) Cells were grown in 1% serum overnight (16 h) and then treated with various concentrations of NVP‐AEW541 (indicated in μm) for 3 h, after which cells were stimulated with IGF‐1 (50 ng/ml) for 15 min before being lysed and subjected to immunoblotting.

In order to assess the therapeutic potential of targeting IGF1R in TGCT, NVP‐AEW541, an IGF1R inhibitor, was used in GI_50_ assays (Figure 4A). The IGF1R inhibitor was effective at inhibiting IGF1R and AKT phosphorylation in response to IGF‐1 stimulation at all concentrations in all cell lines (Figure 4B–D). The most sensitive cell lines were those that had shown high levels of IGF1R phosphorylation in response to IGF stimulus. The seminoma cell line TCAM2 was one of the most resistant lines to NVP‐AEW541 inhibition, consistent with a mild phenotypic response to IGF1R silencing, and the 2102 cell line, which demonstrated the lowest phospho‐AKT levels, was the most resistant.

**Figure 5:**
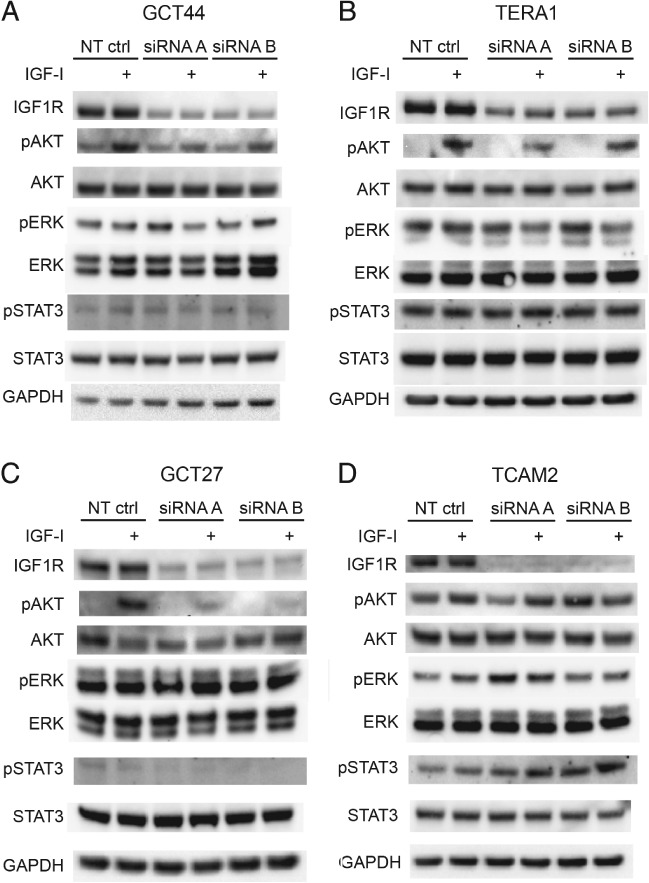
Attenuation of AKT signalling in nonseminoma TGCT cells following IGF1R siRNA treatment. Following 48 h siRNA treatment, the TGCT cell lines GCT44 (A), TERA1 (B), GCT27 (C), and TCAM2 (D) were grown in 1% serum overnight (16 h) and then stimulated with 50 ng/ml IGF‐1 for 15 min before lysis. IGF1R knockdown and AKT, ERK, and STAT3 activation were assessed by immunoblotting of whole cell lysates. Densitometry data corresponding to these blots are shown in the supplementary material, Figure S4.

Sensitivity to NVP‐AEW541 in our cell line panel broadly reflects IGF1R activity, although the GCT27 cell line was moderately sensitive despite having very low levels of phospho‐IGF1R. The low activity of IGF1R in GCT27 may significantly alter downstream signalling. To investigate this possibility, we assessed the effects of silencing IGF1R on signalling pathways downstream of IGF1R in response to IGF‐I stimulation (Figure 5). In cell lines that appear to depend on IGF1R signalling, such as GCT44, and have the highest sensitivity to an IGF1R inhibitor, such as TERA1, siRNA‐mediated downregulation of IGF1R led to decreased phospho‐AKT when stimulated with IGF‐I (Figure 5A, B). This was also the case for the GCT27 line (Figure 5C), whereas phospho‐AKT levels did not alter when IGF1R was silenced in TCAM2 cells, which do not have a high dependence on IGF1R signalling and are more resistant to the inhibitor (Figure 5D). Changes in the levels of phospho‐ERK and phospho‐STAT3 upon IGF1R silencing were not observed.

### IGF1R protein expression is increased in the acquired cisplatin‐resistant TGCT model cell line and IGF1R silencing leads to cisplatin resensitization

**Figure 6:**
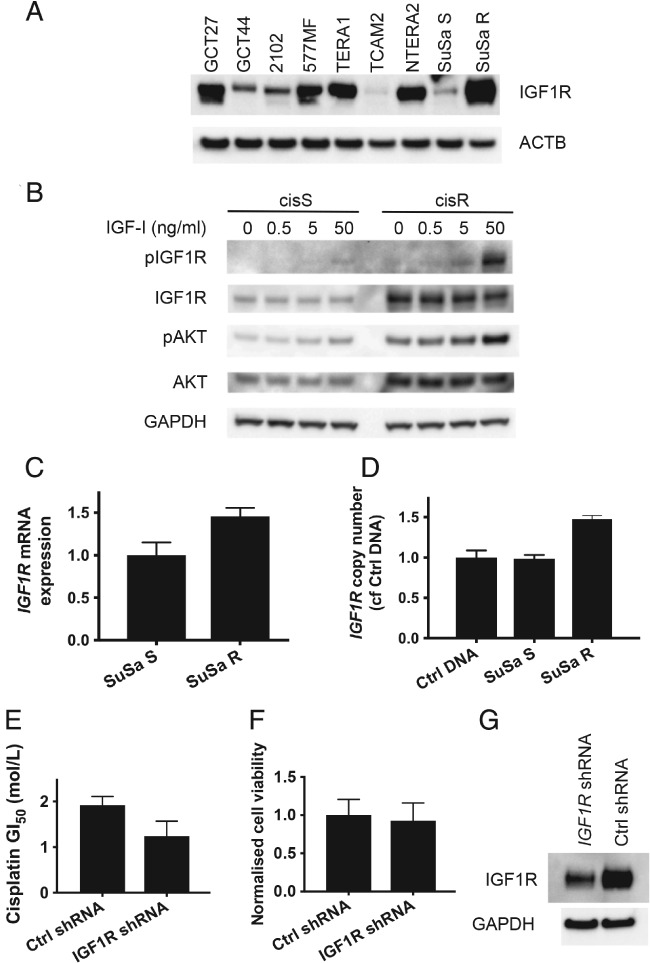
Upregulation of IGF1R protein and pathway activation in SuSa cisplatin‐resistant (cisR) cells. (A) IGF1R and phospho‐AKT are upregulated in the SuSa cisR subline compared with the parental sensitive cells (cisS) and show a hyperactive response to IGF‐1 stimulation. (B) SuSa cisR cells have the highest IGF1R expression across all the cell lines used in the study. IGF1R mRNA expression (C) and copy number (D) are increased in SuSa cisR cells compared with their cisplatin‐sensitive counterpart. (E) Reduced IGF1R expression following shRNA lentiviral transduction results in sensitization of SuSa cisR cells to cisplatin. (F) Cell viability in the absence of cisplatin treatment was measured as part of the experiment shown in E and normalized to the control shRNA‐treated cells. (G) Immunoblot confirming reduced expression in IGF1R shRNA‐treated cells. Densitometry data corresponding to the blots in A and B are shown in the supplementary material, Figure S5.

IGF1R has previously been implicated in cisplatin resistance in ovarian cancer 41. We therefore investigated the SuSa cell line model of cisplatin resistance 35 to determine if IGF1R signalling was enhanced in SuSa cisplatin‐resistant (SuSa cisR) cells compared with the parental cisplatin‐sensitive cells (SuSa cisS). IGF1R protein was upregulated in the resistant cells that increased phospho‐AKT signalling when stimulated with IGF‐1, and had the highest expression of IGF1R of all the cell lines in this study (Figure 6A, B). The resistant subline also had increased basal phospho‐AKT levels compared with the sensitive line (Figure 6B). IGF1R expression and copy number were increased 1.5‐fold in the resistant cells compared with the sensitive line (Figure 6C, D). We transduced SuSa cisR cells with either IGF1R shRNA or control non‐silencing (NS) lentivirus and following selection in puromycin and replating, we exposed cells to various doses of cisplatin. The GI_50_ for cisplatin was reduced in the IGF1R shRNA transduced cells compared with the NS‐treated cells (Figure 6E). This difference in sensitivity was seen, although there was no clear difference in cell viability (Figure 6F). However, despite effective knockdown (Figure 6G), residual expression remained quite high and could explain this. Overall, this provides evidence for IGF1R activity contributing to cisplatin resistance in TGCT.

## Discussion

Here, we have identified active IGF1R signalling and have demonstrated that this plays a key role in maintaining nonseminoma cell viability and can also contribute to cisplatin resistance. Our finding of phosphorylated IGF1R in nonseminoma cells and high IGF1R expression levels in patient samples fits with previous observations noting high serum levels of IGF‐2 and IGFBP2 in nonseminoma patients that reduced after treatment 42. Similarly, the proportion of cleaved IGFBP3 (which promotes proliferation in breast cancer 43) is greater in nonseminoma patients and declines upon response to treatment.

The RTK KIT and the chemokine receptor CXCR4 are involved in the survival and migration of TGCTs and also PGCs, the likely precursor cells of TGCT 1, 19, 36. IGF1R signalling is also required for the development of PGCs and gonocytes. Zebrafish models have shown that the IGF1Rb isoform is required for proper migration and maintenance of PGC numbers until they reach the genital ridge 2. Notably, IGF signalling is also required for maintaining the germ cell population in postnatal mice with unilateral undescended testis in both the affected testis and the contralateral descended testis 44. Blockade of IGF1R signalling or the downstream PI3K pathway resulted in loss of pluripotency in murine neonatal spermatogonial stem cells which is maintained by IGF1 produced by the Leydig cells *in vivo* 45. Together with our data, this points to TGCT retaining or recapitulating the IGF1R signalling observed in the germ cell lineage.

Our data demonstrating that activation of IGF1R signalling contributes to nonseminoma cell survival are similar to the functional roles reported for IGF1R in other cancers, including prostate, breast, and colorectal cancers 46, 47, 48. Although IGF1R kinase activity can initiate several major signalling cascades including RAS/RAF/MEK/ERK, PI3K/AKT, and STAT3, we did not observe perturbation of ERK or STAT3 signalling in response to silencing IGF1R in TGCT cell lines. Cell lines demonstrating high phospho‐AKT in response to IGF1 stimulation, which subsequently decreased upon IGF1R silencing, were the most sensitive to chemical IGF1R inhibition and silencing. In contrast, the two least sensitive cell lines either had very low phospho‐AKT levels (in cell line 2102, levels were undetectable) or did not alter phospho‐AKT when IGF1R was downregulated (TCAM2). In addition, the GCT27 cell line, which had little active IGF1R, nonetheless exhibited decreased AKT signalling when IGF1R expression was reduced and was moderately sensitive to the IGF1R inhibitor, as well as demonstrating an intermediate proliferative response to shRNA‐mediated silencing. Therefore, AKT signalling appears to be a key response to IGF1R activation in TGCT cells. This is consistent with data for rhabdomyosarcomas, where sensitivity to IGF1R inhibitors was reversed by constitutively active AKT and, conversely, progression of *in vivo* tumour models was associated with AKT reactivation 49.

To date, few studies have evaluated the roles of RTKs in nonseminomas. A patient with cisplatin‐resistant TGCT responded to trastuzumab, suggesting that ERBB2 may be an effective target in TGCT 50. However, subsequent immunohistochemistry shows that ERBB2 expression is infrequent in TGCT and is not associated with poorer overall survival 51, 52, 53, 54. ERBB3 has been reported as highly expressed in TGCT tumours with signal activation dependent on ERBB2 activation with which it forms heterodimers. Use of lapatinib, a dual ERBB1 and ERBB2 inhibitor, reduced tumour growth in an orthotopic animal model, suggesting that it may be of benefit to a subset of TGCT patients 55. EGFR is expressed by 21–89% of nonseminoma samples 51, 52, 56, 57 and PDGRβ has been linked to cisplatin resistance 58. Recently, ERBB2, EGFR, IGF1R, and other kinases have been investigated in cell lines 59. Consistent with these studies, our phospho‐RTK array screen also identified EGFR and PDGFRβ as the fifth and sixth most active RTKs in TGCT cell lines, with ERBB receptors ranking lower. Interestingly, activation of additional RTKs was identified in our screen including TYRO3 and EPHB2. Their role in TGCT remains to be elucidated.

Unlike many solid tumours, TGCT cells readily undergo apoptosis in response to DNA‐damaging agents such as cisplatin via a p53‐dependent pathway. The p53 transcriptional response to DNA damage appears intact but leads to apoptosis in preference to cell cycle arrest, due in part to very low levels of p21 in TGCT cells 60. In cases of cisplatin resistance, AKT has been shown to be essential for high cytoplasmic p21 expression, which when reversed by AKT inhibition restores cisplatin sensitivity 61. *AKT1* mutations have recently been identified exclusively in cisplatin‐resistant germ cell tumours 62, and copy number gain and concomitant overexpression of AKT1 were a frequent event in a study of intracranial germ cell tumours, which although clinically and histologically similar to gonadal germ cell tumours, are more likely to be refractory to treatment 63. Phospho‐AKT levels are significantly higher in cisplatin‐resistant TGCT cells compared with sensitive or untreated tumours 58. This is consistent with our finding that in one TGCT model of acquired cisplatin resistance, *IGF1R* copy number and mRNA expression were increased in the cells with cisplatin resistance, accompanied by elevated IGF1R and phospho‐AKT protein levels. Importantly, reducing IGF1R expression in the resistant subline resensitized cells to cisplatin, showing that upregulation of IGF1R can contribute to cisplatin resistance in TGCT.

Single‐agent clinical trials employing IGF1R inhibitors have been largely disappointing, due to a lack of both wide‐scale efficacy and accurate predictive biomarkers of response. There is conflicting evidence as to whether expression of IGF1R itself, as opposed to activated IGF1R, identifies patients as suitable for IGF1R‐targeted therapy 49, 64, 65, 66. Our data identify AKT pathway activation in response to IGF1R signalling as a key factor. IGF1R inhibition has resulted in enhanced sensitivity to general cytotoxic and radiation‐based therapies in other cancers 67, 68, 69, and recent results for TGCT cell lines show positive but mixed results for cisplatin combined with mTOR, EGFR, and IGF1R inhibitors 59. Together with our results, this raises the possibility that combination of IGF1R inhibition with chemotherapy could promote resensitization to treatment in chemo‐resistant disease.

## Author contributions statement

JS, NCG, AM, and JMS designed the study. JS, NCG, KRT, and JR carried out and interpreted experiments. JS, NCG, SDP, and KT analysed data. JS, NCG, BMS, RAH, and JMS were involved in data interpretation. JS, NCG, DCG, and JMS wrote the manuscript with contributions and approval from all authors.

**SUPPLEMENTARY MATERIAL ONLINE**

**Supplementary figure legends**

**Figure S1.** Phosphorylated receptor tyrosine kinase levels in TGCT cell lines

**Figure S2.** shRNA‐mediated silencing of IGF1R results in apoptosis

**Figure S3.** Densitometry data for immunoblots shown in Figure 1

**Figure S4.** Densitometry data for immunoblots shown in Figure 5

**Figure S5.** Densitometry data for immunoblots shown in Figure 6

**Table S1.** Histological subtypes of primary TGCT samples

**Table S2.** IGF1R TMA IHC staining intensity scores

## Supporting information


**Supplementary figure legends**

**Figure S1. Phosphorylated receptor tyrosine kinase levels in TGCT cell lines.** Whole lysates from TGCT cell lines were incubated onto Phospho‐RTK array membranes spotted with 49 different RTK antibodies in duplicate and phosphorylation levels detected by probing with an anti‐phosphotyrosine antibody. TGCT cell lines (A) GCT27, (B) GCT44, (C) 2102, (D) 577MF, (E) TERA1, (F) TCAM2, and (G) NTERA2 were used. The positions of the top six ranking kinases are indicated by a coloured line beneath the duplicate signals on the membrane arrays.

**Figure S2. shRNA‐mediated silencing of IGF1R results in apoptosis.** (A) Reduction of IGF1R protein following siRNA treatment is shown by immunoblotting. (B) Reduction of IGF1R protein following 6 days post‐shRNA lentiviral transduction (including selection) is shown by immunoblotting. (C) IGF1R shRNA‐treated NTERA2 cells show a much higher degree of apoptosis than control shRNA‐treated cells 8 days post‐transduction as assessed by caspase 3/7 activity.

**Figure S3. Densitometry data for immunoblots shown in Figure** 1. Densitometry data are shown for blots in Figure 1B, C. The title of each graph indicates which bands have been quantified and what they have been normalized to.

**Figure S4. Densitometry data for immunoblots shown in Figure** 5. Densitometry data are shown for selected blots in Figure 5. The title of each graph indicates which bands have been quantified and what they have been normalized to.

**Figure S5. Densitometry data for immunoblots shown in Figure** 6. Densitometry data are shown for blots in Figure 6A, B. The title of each graph indicates which bands have been quantified and what they have been normalized to.

**Table S1.** Histological subtypes of primary TGCT samples

**Table S2.** IGF1R TMA IHC staining intensity scores
