# Supplementary material for: IGF1R signalling in testicular germ cell tumour cells impacts on cell survival and acquired cisplatin resistance
Source: J Pathol. 2018 Jan 10;244(2):242–53. doi: 10.1002/path.5008 (PMC5817239; doi:10.1002/path.5008)
Supplement: Supplementary file 1 — Supplementary figure legends [file PATH-244-242-s002.doc]

**Supplementary figure legends**

**Figure S1. Phosphorylated receptor tyrosine kinase levels in TGCT cell lines.** Whole lysates from TGCT cell lines were incubated onto Phospho-RTK array membranes spotted with 49 different RTK antibodies in duplicate and phosphorylation levels detected by probing with an anti-phosphotyrosine antibody. TGCT cell lines (A) GCT27, (B) GCT44, (C) 2102, (D) 577MF, (E) TERA1, (F) TCAM2, and (G) NTERA2 were used. The positions of the top six ranking kinases are indicated by a coloured line beneath the duplicate signals on the membrane arrays.

**Figure S2**. **shRNA-mediated silencing of IGF1R results in apoptosis.** (A) Reduction of IGF1R protein following siRNA treatment is shown by immunoblotting. (B) Reduction of IGF1R protein following 6 days post-shRNA lentiviral transduction (including selection) is shown by immunoblotting. (C) IGF1R shRNA-treated NTERA2 cells show a much higher degree of apoptosis than control shRNA-treated cells 8 days post-transduction as assessed by caspase 3/7 activity.

**Figure S3. Densitometry data for immunoblots shown in Figure 1.** Densitometry data are shown for blots in Figure 1B, C. The title of each graph indicates which bands have been quantified and what they have been normalized to.

**Figure S4. Densitometry data for immunoblots shown in Figure 5.** Densitometry data are shown for selected blots in Figure 5. The title of each graph indicates which bands have been quantified and what they have been normalized to.

**Figure S5. Densitometry data for immunoblots shown in Figure 6.** Densitometry data are shown for blots in Figure 6A, B. The title of each graph indicates which bands have been quantified and what they have been normalized to.
