# Supplementary material for: IGF1R signalling in testicular germ cell tumour cells impacts on cell survival and acquired cisplatin resistance
Source: J Pathol. 2018 Jan 10;244(2):242–53. doi: 10.1002/path.5008 (PMC5817239; doi:10.1002/path.5008)
Supplement: Supplementary file 7 — Table S1. Histological subtypes of primary TGCT samples [file PATH-244-242-s006.docx]

**Table S1.** Histological subtypes of primary TGCT samples

| **Sample** | **Histology** |
| --- | --- |
| NS1 | MTD |
| NS2 | MTU |
| NS3 | MTU |
| NS4 | MTI/NS |
| NS5 | MTD |
| NS6 | MTU/MTD |
| NS7 | MTD/NS |
| NS8 | MTD |
| NS9 | MTU |
| NS10 | MTD |
| NS11 | MTI/NS |
| NS12 | MTU/NS |
| NS13 | MTD |
| NS14 | MTI/YS |
| NS15 | MTD |
| NS16 | MTD/NS |
| NS17 | MTU/YS/NS |
| NS19 | MTD |
| NS20 | MTD |

NS: nonseminoma; MTU: malignant teratoma undifferentiated; MTI: malignant teratoma intermediate differentiation; MTD: malignant teratoma differentiated; YS: yolk sac tumour.
