# Supplementary material for: IGF1R signalling in testicular germ cell tumour cells impacts on cell survival and acquired cisplatin resistance
Source: J Pathol. 2018 Jan 10;244(2):242–53. doi: 10.1002/path.5008 (PMC5817239; doi:10.1002/path.5008)
Supplement: Supplementary file 8 — Table S2. IGF1R TMA IHC staining intensity scores [file PATH-244-242-s008.docx]

**Table S2.** IGF1R TMA IHC staining intensity scores

| **Intensity score** | **Nonseminomas** | **Seminomas** | **Total** |
| --- | --- | --- | --- |
| 0 | 75 | 3 | 78 |
| 1 | 27 | 7 | 35 |
| 2 | 37 | 3 | 39 |
| 3 | 9 | 3 | 12 |
| Total | 148 | 16 | 164 |
